# Genomic insights into ST85 and ST158 belonging to recently emerged global clones of multidrug-resistant *Acinetobacter baumannii* isolates from Egypt: in vitro assessment of repurposed drug–antibiotic combinations

**DOI:** 10.1186/s12941-025-00829-0

**Published:** 2025-11-15

**Authors:** Mona S. El Far, Mervat A. Kassem, Eva A. Edward, Benjamin A. Evans, Dave J. Baker, Azza S. Zakaria

**Affiliations:** 1 Department of Microbiology and Immunology, Faculty of Pharmacy, Alexandria University, El-Khartoom Square, Azarita, Alexandria, Egypt; 2 Norwich Medical School, University of East Anglia, Norwich, UK; 3 Quadram Institute Bioscience, Norwich, UK

**Keywords:** *Acinetobacter baumannii*, Multidrug-resistant, ST85, ST158, WGS, Phylogenetic analysis, Checkerboard assay, Repurposed drugs, *N*-Acetylcysteine

## Abstract

**Background:**

The strikingly rapid increase in multidrug-resistant *Acinetobacter baumannii* (MDRAB) incidence rates represents a major challenge in healthcare settings. This is due to the limitation of the currently available treatment options to combat tenacious *A. baumannii* infections. MDRAB isolates belonging to recently emerged global clones GC9 and GC10 are on the rise, especially in the Middle East and Africa, which warrants a thorough investigation of these global clones.

**Methods:**

Thirteen *A. baumannii* isolates belonging to less well-studied global clones were selected from 46 isolates collected in Alexandria, Egypt, after determining their clone using MLST. Susceptibility to multiple antibiotic classes was determined by the Kirby-Bauer disk diffusion method. Testing of carbapenemase activity and selected virulence phenotypes was done. Whole genome sequencing, phylogenetic analysis, and molecular characterization of the resistance and virulence genotypes were performed. Checkerboard assay was employed for testing the combination of each of ciclopirox and *N*-acetylcysteine (NAC), as potential repurposed drugs, with each of meropenem and levofloxacin antibiotics against MDRAB isolates.

**Results:**

All the isolates displayed multidrug resistance and were carbapenemase-positive. One isolate showed strong biofilm formation, whereas 4 and 8 isolates were moderate and weak biofilm formers, respectively. Twelve out of thirteen isolates were positive twitchers. The isolates showed moderate phospholipase and strong protease activities. However, low phospholipase production was detected in one isolate. The genomic analysis revealed that 3 and 10 isolates belonged to ST85 (GC9) and ST158 (GC10), respectively. All 13 isolates harbored multiple resistance genes including *oxa23* and carried an RP-T1 rep type plasmid. Phylogenetic analysis demonstrated that the isolates were clustered together forming subclades with others from Alexandria/Egypt. The AbGRI3-2 resistance island (RI) was detected in ST158 isolates carrying R3-T60 rep type and 9 antibiotic resistance genes. The combination of NAC with each of meropenem or levofloxacin showed a synergistic action against 3 and one isolate(s), respectively, using the checkerboard assay.

**Conclusion:**

The current study provides an in-depth characterization of the collected MDRAB isolates from the global clones GC9 and GC10. The endemicity of these clones necessitates strategies to mitigate ongoing MDRAB outbreaks in countries like Egypt. Combination of NAC with meropenem or levofloxacin represents a promising treatment option against the newly emerged global clones that needs further in vivo testing.

**Supplementary Information:**

The online version contains supplementary material available at 10.1186/s12941-025-00829-0.

## Introduction

*Acinetobacter baumannii* is a Gram-negative, coccobacillus, aerobic, non-fermentative ESKAPE pathogen commonly found in healthcare environments, particularly intensive care units [1]. It persists in hospital settings due to the organism’s resistance to desiccation and its ability to form biofilms on surfaces and medical equipment [2, 3]. This opportunistic pathogen is responsible for a wide array of infections, especially among immunocompromised patients, such as pneumonia, meningitis, bacteremia, wound infections, respiratory tract, and urinary tract infections [1, 4]. The last decade has witnessed a remarkable global increase in the occurrence of such infections with a mortality rate ranging between 30 and 75% [1].

*A. baumannii* is characterized by its rapid development of multidrug resistance enabled by multiple mobile genetic elements (MGEs) and high genomic plasticity [5, 6]. Multidrug-resistant *A. baumannii* (MDRAB) strains showing carbapenem resistance have been reported worldwide which raises alarming concerns about the limited availability of therapeutic options [7]. Owing to the clonal expansion of carbapenem-resistant *A. baumannii* (CRAB) strains, CRAB infection rates exceeded 70% in some regions worldwide [8]. This worrisome trajectory has resulted in the World Health Organization (WHO) listing CRAB among the top priority pathogens for the development of novel treatment options [9].

MDRAB infections, including CRAB infections, represent a heavy burden in several low and middle-income countries [10]. Egypt is considered among the countries that are gravely impacted by CRAB and MDRAB infections [11–13]. In Egypt, the prevalence rates of MDRAB and CRAB, in the period from 2004 to 2020, were found to be between 30 and 100% and 26.6–100% of *A. baumannii* clinical isolates, respectively [14].

As revealed by epidemiological and molecular studies, nine major global clones (GC1-9) of *A. baumannii* have been distinguished. The most globally disseminated clone is GC2, followed by GC1, these are responsible for most CRAB outbreaks reported in hospital settings worldwide [11, 15]. Nonetheless, owing to the dynamic nature and genomic plasticity of *A. baumannii*, the emergence and propagation of antimicrobial resistance (AMR) phenotypes in other non-major GCs such as GC9 have been reported [11, 16]. GC9 is one of the most recent international high-risk global clones and includes strains belonging to sequence type 85 (ST85), according to the Pasteur multi-locus sequence typing (MLST) scheme, and harboring class-B metallo-beta-lactamases (*bla*_NDM_) [11, 17]. Although it is globally distributed, this genetic lineage was reported to be more specifically prevalent in the Middle East and North Africa [17]. Moreover, *A. baumannii* isolates belonging to Pasteur MLST ST158 represent a novel global clone designated GC10 [8]. This clonal lineage has been previously detected in several countries in the Middle East, North Africa, and the Mediterranean region including Egypt, Jordan, Lebanon, Iraq, Kuwait, Saudi Arabia, Tunisia, and Turkey [8, 12]. Notably, *oxaAb*(65) and *bla*_ADC−117_ were considered genetic signatures that characterize ST158 isolates. Also, most of these isolates harbored the *oxa23* carbapenemase gene [8].

Colistin and tigecycline are considered viable treatment options for infections caused by MDRAB, including CRAB [18]. However, the use of these antibiotics has been hindered by the severe physical adverse effects, such as nephrotoxicity or neurotoxicity in the case of colistin [19] and reported coagulopathy events associated with tigecycline [20]. Moreover, the emergence of extensive drug-resistant (XDR) or pan-drug-resistant (PDR) strains has eroded the effectiveness of these drugs and rendered it an urgent priority to develop novel molecules [21]. The conventional strategy of drug development is expensive and time-consuming. Thus, “Drug repurposing”, a recently proposed approach, offers a promising cost-effective, and time-saving alternative [22]. Recent studies have focused on exploring the efficacy of the combination of antibiotics and repurposed drugs to combat MDRAB, especially CRAB [18]. A wide range of FDA-approved drugs belonging to multiple pharmacological classes have been repurposed including antifungals (ciclopirox), and mucolytics (NAC) [23, 24].

Many studies have investigated resistance and virulence profiles of MDRAB across the world. However, there are few in-depth studies in regions such as the Middle East or Africa. These are needed for a better and more comprehensive understanding of MDRAB and CRAB belonging to the most recent global clones GC9 and GC10. This study aimed to characterize the resistance and virulence profiles of MDRAB, belonging to ST85 (GC9) and ST158 (GC10), collected from Alexandria, Egypt. Phylogenetic and comparative genomic analyses have been conducted to investigate the relatedness of the tested isolates to other previously collected *A. baumannii* isolates from the Middle East or Africa. Furthermore, the in vitro evaluation of the combination of either ciclopirox or NAC, as potential repurposed drugs, with each of meropenem and levofloxacin antibiotics against MDRAB clinical isolates has been performed.

## Methods

### Collection and identification of bacterial isolates

**Fig. 1:**
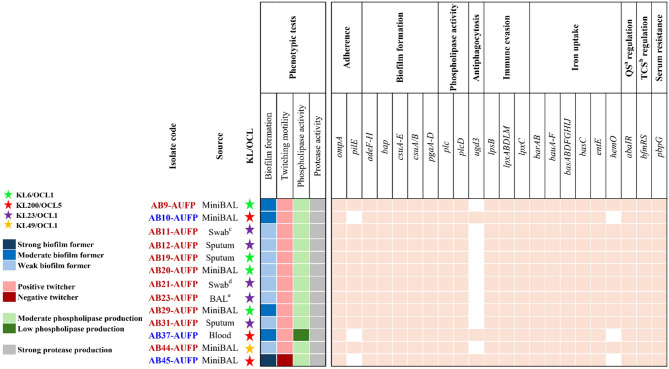
Illustration of diverse sources of isolation and KL/OCL patterns of *A. baumannii* clinical isolates, along with a heatmap showing selected phenotypic virulence traits as well as virulence determinants of the tested strains. Red labeling refers to ST158 (GC10) isolates, while blue labels indicate the isolates belonging to ST85 (GC9). Pastel- and white-colored squares in the heatmap represent the presence and absence of virulence genes, respectively. ^a^Quorum sensing, ^b^Two-component system, ^c^Swab from a chest wound, ^d^Swab from leg lesions, ^e^Bronchoalveolar lavage

Forty-six non-duplicate *Acinetobacter* clinical isolates, obtained from different hospitals, were collected from Mabaret Al-Asafra lab, Alexandria, Egypt, from March 2023 to September 2023. Out of this collection, 13 *Acinetobacter* strains, belonging to less well-studied global clones, were selected in this study after performing MLST to determine their clone (https://github.com/tseemann/mlst). They were isolated from various clinical specimens (Fig. 1). VITEK^®^ 2 Compact System (bioMérieux, Marcy-L’Etoile, France) was used with a GN identification card, in compliance with the manufacturer’s instructions, to identify the collected isolates to the species level. For further confirmation of the isolates’ identity as *A. baumannii*, the *oxaAB* gene was amplified using polymerase chain reaction (PCR) [25]. Frozen stocks of the isolates were stored in Luria-Bertani broth (LB, tryptone 1%, yeast extract 0.5%, and sodium chloride 1%) containing 20% glycerol at − 80 °C for long-term preservation.

### Antimicrobial susceptibility testing

The susceptibility of *A. baumannii* isolates to 19 antimicrobial agents was determined by the Kirby-Bauer disk diffusion method, according to the Clinical Laboratory Standards Institute (CLSI, 2021) guidelines, using piperacillin, piperacillin/tazobactam, ampicillin/sulbactam, ticarcillin/clavulanate, ceftazidime, ceftriaxone, cefotaxime, cefepime, imipenem, meropenem, amikacin, gentamicin, tobramycin, tetracycline, doxycycline, minocycline, ciprofloxacin, levofloxacin, and sulfamethoxazole/trimethoprim discs (Microxpress^®^, Tulip Diagnostics Ltd, India). The results were interpreted following the CLSI breakpoints [26]. Determination of colistin susceptibility was done using VITEK^®^ 2 Compact system with Advanced Expert System (AES) software (bioMérieux, Marcy-L’Etoile, France) in conjunction with an Ast N222 sensitivity card, as specified by the manufacturer.

### Phenotypic detection of carbapenemase activity

#### Triton Hodge test (THT)

THT method, an improved version of the modified Hodge test, was applied to detect membrane-bound carbapenemases. About 50 µL of pure Triton X-100 (BIOFROXX, Germany) were dropped at the center of a Mueller Hinton agar (MHA) plate and then spread on the whole agar surface using sterile swabs. To ensure complete absorbance of the reagent, the plate was left unmoved for about 10 min. An inoculum of *E. coli* NCTC 10418, as an indicator strain, was adjusted to a 0.5 McFarland turbidity standard, 1:10 diluted in saline, then used to swab the surface of the MHA plate. After keeping the plate for 10 min at room temperature, a disk containing meropenem (10 µg) was applied on the agar surface. Then, 3 to 5 colonies of the tested isolate were inoculated on the plate, using a sterile swab, out from the edge of the disk in a straight line [27, 28]. After incubation at 37 °C for 18–24 h, the plate was inspected for a clover leaf-type indentation in the carbapenem disc inhibition zone at the site of intersection of the indicator strain and the tested clinical isolate [29].

### Phenotypic detection of selected virulence factors

#### Biofilm formation assay

The overnight culture of each tested isolate was centrifuged in 5 mL LB broth supplemented with glucose (0.5%) [30] at 7000 rpm for 10 min and the supernatant was then discarded. The obtained bacterial pellet was suspended in normal saline and adjusted to 0.5 McFarland standard [31]. After 1:20 dilution of the bacterial suspension in the aforementioned media, 200 µL were inoculated in a sterile 96-well flat-bottomed microtiter plate and then incubated at 37 °C for 24 h. The wells were rinsed twice with 200 µL of phosphate-buffered saline (PBS), air-dried for 15 min, and stained with 200 µL of crystal violet solution (0.1% v/v) for 20 min. After washing with PBS, destaining was performed using 200 µL of acetic acid (33% v/v) and the plates were incubated for 15 min at 37 °C before measuring the optical density at 630 nm using a microtiter plate reader (Biotek, USA). Uninoculated wells and wells containing a bacterial culture of *A. baumannii* ATCC19606 served as negative and positive controls, respectively. Every data point was the average of absorbance readings of triplicate wells (OD_S_). The OD cut-off value (ODc) was calculated as three standard deviations above the mean absorbance of the negative control. The tested strains were categorized as strong (OD_S_ > 4 × ODc); moderate (2 × ODc < OD_S_ ≤ 4 × ODc); weak (ODc < OD_S_ ≤ 2 × ODc); or no (OD_S_ ≤ ODc) biofilm producers. The experiment was performed twice for each isolate [32].

#### Twitching motility assay

Motility assay was conducted using LB-no salt agar medium (yeast extract 5 g/L; tryptone 10 g/L; and 1% agar-agar) [33]. To allow the bacterial spread at the interphase between the medium and the bottom of the plate, fresh cultures of the tested isolates were inoculated by stabbing till the agar bottom, then the plates were incubated for 48 h at 37 °C. For visualization, after discarding the agar, the plates were stained using 0.2% crystal violet [34]. The assay was repeated two times. Isolates showing a zone greater than 10 mm surrounding the inoculation site were recognized as positive twitchers [35].

#### Phospholipase C activity

For each isolate, an inoculum of 2 µL of overnight culture in nutrient broth (a 0.5 McFarland equivalent) was inoculated by dropping onto 2 sectors of a modified egg yolk agar medium (nutrient agar supplemented with 5% egg yolk and 0.11% calcium chloride) [36], then the plates were incubated for 48 h at 37 °C. The formation of a white dense precipitation zone extending from the colony edge indicated a positive phospholipase C activity. The zone of phospholipase activity (Pz value) was determined as the ratio of the colony diameter to the total diameter of the colony plus the precipitation zone. The strains were classified as follows: those with high phospholipase activity (Pz < 0.5); moderate activity (Pz = 0.5–0.69); low activity (Pz = 0.99–0.7); and no activity (Pz = 1) [37].

#### Protease activity

Screening for protease activity was done using bovine serum albumin (BSA) medium (2% dextrose, 0.1% KH_2_PO_4_, 0.05% MgSO_4_, 2% agar, and 1% bovine serum albumin (Biowest, France)) as described by Mohan and Ballal [38]. For each isolate, 5 mL LB broth was inoculated by 0.5 mL of overnight culture in LB broth (a 0.5 McFarland equivalent) and incubated for 18 h at 37 °C. After centrifugation for 15 min at 8500 g, 100 µL of the supernatant were added to formerly punched cups in the BSA medium, then the plates were incubated overnight at 37 °C [37]. The precipitation zone (Pz) value was determined as the ratio of the cup diameter to the diameter of the cup plus that of the precipitation zone. According to the production of protease enzymes, the tested strains were categorized as follows: high producers (Pz = 0.35–0.5); moderate producers (Pz = 0.51–0.74); low producers (Pz = 0.75–0.9); and non-producers (Pz = 1). Each sample was dropped in 2 cups/plate and the average value of two measurements was considered [39].

### DNA extraction and whole genome sequencing (WGS)

#### DNA extraction and sequencing

Extraction and purification of bacterial DNA was performed using GeneJET Genomic DNA Purification Kit (Thermo Fisher Scientific, Vilnius, Lithuania), as specified by the manufacturer. WGS of the thirteen collected *A. baumannii* clinical isolates was conducted by the sequencing department at the Quadram Research Institute, Norwich, UK. The purity of DNA was evaluated by measuring the 260/280 ratio on a NanoDrop One^C^ Spectrophotometer (Thermo Fisher Scientific). DNA was quantified using the Promega QuantiFluor^®^ dsDNA System (Catalogue No. E2670) and run on a GloMax^®^ Discover Microplate Reader. Short-read sequencing was performed on an Illumina Nextseq2000 instrument. Three isolates, AB37-AUFP (as a representative for ST85) as well as AB11-AUFP and AB44-AUFP (as representatives for ST158), were selected for long-read sequencing. This was performed with a promethION R10 flow cell on a P2 Solo in accordance with Oxford Nanopore Technologies protocol (SQK-LSK114). Base calling was carried out using Guppy [5+] (Oxford Nanopore Technologies).

#### Read quality check and assembly

Raw short reads were trimmed using Trimmomatic v0.39 [40], and the quality of reads was checked utilizing FastQC v0.12.1 [41]. Trimmed short reads were de novo assembled through Unicycler v0.5.0 [42]. Hybracter v0.7.3 [43] was employed for the reads’ quality control, removing unwanted contaminants and adapters, along with long-read assembly polished with long and short-reads of the three selected isolates: AB11-AUFP, AB37-AUFP, and AB44-AUFP. The quality of the obtained assemblies was checked with CheckM v1.0.18 [44], and only genomes showing a completeness threshold of > 95% and a contamination threshold of < 5% were retained for further analysis.

#### Identifying genetic features

MLST of the tested isolates was ascertained based on the Pasteur typing scheme through the MLST tool v2.23.0. RGI v6.0.3 and ABRicate v1.0.1 (https://github.com/tseemann/abricate) were employed for detecting antibiotic resistance genes relying on the Comprehensive Antibiotic Resistance Database (CARD) [45]. The BLAST function on the Beta-Lactamase Database [46] was utilized for confirming all *oxaAb* alleles. Mutations in quinolone resistance-determining regions (QRDRs) of *gyrA* and *parC* genes were analyzed [11]. The amino acid sequences encoded by *gyrA* and *parC* genes from the tested isolates were aligned with the corresponding sequences in *A. baumannii* ATCC 19,606, utilizing the Clustal Omega v1.2.4 multiple sequence alignment (MSA) service available at the EMBL’s European Bioinformatics Institute (EMBL-EBI) website (accessed 15 July 2024) [47], and visualized using the MSA viewer tool Jalview v2.11.3.3 [48]. Virulence factors were screened through the Virulence Factors Database (VFDB) search tool [49]. For *Acinetobacter* surface polysaccharide locus typing, Kaptive v3.0.0b5 was utilized based on a reference database for both capsule synthesis loci (K, or KL-loci) and lipooligosaccharide outer core loci (OCL) [50, 51]. Plasmids were identified by Mobsuite v3.1.8 [52] and Plasmer [53]. Plassembler, incorporated in the Hybracter pipeline, was employed to recover plasmids from long-read assemblies [54]. Detection of replicase genes and plasmids’ classification were performed using the *Acinetobacter* Plasmid Typing database against the draft genome as previously described [55, 56]. Annotation of draft genomes was done using Prokka v1.14.6 [57] and the RAST web server [58] while that of long-read assemblies was performed through the NCBI Prokaryotic Genome Annotation Pipeline [59]. Mobile elements were predicted using MobileElementFinder v1.0.3 [60]. Prediction of resistance islands in the complete genomes of AB11-AUFP, AB37-AUFP, and AB44-AUFP isolates was conducted using the IslandViewer4 web tool (http://www.pathogenomics.sfu.ca/islandviewer/) [61]. A circular comparison map of the complete chromosomes of AB11-AUFP, AB37-AUFP, and AB44-AUFP, together with other similar chromosomes of strains in the Middle Eastern or African region, was created using the online software Proksee.ca (accessed 1 July 2024) [62]. The analysis of the genomic structure of the chromosomal resistance island in the isolates AB11-AUFP and AB44-AUFP, and its comparison to islands carried on complete chromosomes that are deposited in the NCBI database and showing more than 95% of nucleotide identity, was done using Clinker [63]. Proksee software was used for the schematic mapping of the complete plasmid sequences pAB11-AUFP1, pAB37-AUFP1, and pAB44-AUFP1 with other reported similar plasmids.

#### Phylogenetic analysis

The PubMed database was searched using keywords of [(*Acinetobacter baumannii*) AND (ST85) OR (ST158)]. Genomes of *A. baumannii* strains belonging to ST85 or ST158 and detected in African or Middle Eastern countries were downloaded and included in subsequent analyses. In addition, genomes of ST85 or ST158 deposited in the PubMLST database, which were collected from Middle East or Africa regions, were also involved in the phylogenetic analysis. All genomes were annotated utilizing Prokka v1.14.6 [57]. Panaroo v1.5.0 [64] was used to determine the core-genome content of the strains included in the analysis. The phylogeny was predicted through a maximum likelihood tree employing IQ-TREE v2.2.6 [65], and the tree was visualized in iTOL v6 (https://itol.embl.de/).

### The combined activity of repurposed drugs and antibiotics

The combined effect of selected repurposed drugs, ciclopirox or *N*-acetylcysteine, with each of meropenem or levofloxacin, was determined by the previously described simplified checkerboard method performed in a 96-well microtiter plate [66]. A bacterial inoculum with adjusted turbidity of 0.5 McFarland was 100-fold diluted in double strength cation adjusted Mueller–Hinton broth (HiMedia, Mumbai, India). A volume of 100 µL of the tested isolate was added to an equivalent volume of the tested antibiotic/drug combination, at various concentrations, in the checkerboard plates. Positive and negative controls were also included in the experiment. After overnight incubation at 37 °C, the fractional inhibitory concentration index (FICI) was calculated according to the following equation:

$$\begin{aligned} {\text{FICI}} =\frac{{{\text{MIC}}\;{\text{of}}\;{\text{antibiotic}}\;{\text{in}}\;{\text{combination}}}}{{{\text{MIC}}\;{\text{of}}\;{\text{antibiotic}}\;{\text{alone}}}}  + \frac{{{\text{MIC}}\;{\text{of}}\;{\text{repurposed}}\;{\text{drug}}\;{\text{in}}\;{\text{combination}}}}{{{\text{MIC}}\;{\text{of}}\;{\text{repurposed}}\;{\text{drug}}\;{\text{alone}}}} \end{aligned}$$  

The efficacy of the examined combinations was interpreted as follows: FICI ≤ 0.5; synergy, 0.5 < FICI < 1; partial synergy, FICI = 1; additivity, 1 < FICI < 4; indifference, and FICI ≥ 4; antagonism [67].

## Results

### Identification of clinical isolates

Thirteen isolates of *A. baumannii*, belonging to less well-studied global clones (Fig. 1), were identified. These isolates belonged to *A. baumannii* complex as identified by the Vitek 2 compact system. The *oxaAB* gene was detected in all the isolates confirming the identification of *A. baumannii* as the species. The isolates were collected from various clinical specimens as follows: mini-bronchoalveolar lavage (MiniBAL) (*n* = 6), sputum (*n* = 3), swab from a chest wound (*n* = 1), swab from leg lesions (*n* = 1), bronchoalveolar lavage (BAL) (*n* = 1), and blood (*n* = 1) (Fig. 1).

### Phenotypic detection of selected virulence features

Isolates were tested for selected virulence factors namely biofilm formation, twitching motility, phospholipase production, and protease activity. Only 1 isolate (AB45-AUFP) showed strong biofilm formation, whereas 4 and 8 isolates were moderate and weak biofilm formers, respectively. Moreover, all except one isolate were positive twitchers, with the diameters of the twitching zones ranging between 11 mm (for AB11-AUFP) and 58 mm (for AB37-AUFP), except AB45-AUFP which was considered a negative twitcher with a twitching zone diameter less than 10 mm. Additionally, all the isolates showed moderate phospholipase and strong protease activities, however, low production of phospholipase enzyme was detected in the case of AB37-AUFP (Fig. 1).

### Molecular typing and genomic investigation of virulence determinants

The MLST of the tested isolates was determined according to the Institut Pasteur scheme; 3 clinical isolates (AB10-AUFP, AB37-AUFP, and AB45-AUFP) belonged to ST85 (GC9), while the rest of the isolates were related to ST158 (GC10).

Capsule synthesis loci (K, or KL-loci) and lipooligosaccharide outer core loci (OCL) are important virulence features in *A. baumannii* that can be employed for typing purposes [17]. In this study, the KL200/OCL5 pattern was exclusively present in the 3 clinical isolates related to ST85 (GC9). KL6/OCL1 and KL23/OCL1 patterns were found in 4 and 5 of the isolates belonging to ST158 (GC10), respectively, while the KL49/OCL1 pattern was detected in the remaining isolate (AB44-AUFP) (Fig. 1).

Virulome analysis of the tested isolates showed the presence of various virulence determinants that are responsible for multiple virulence factors. Genes that are responsible for adherence (*ompA*), biofilm formation (*adeF-H*, *bap*, *csuA/B*, *csuA-E*, *pgaA-D*), phospholipase activity (*plc*,* plcD*), immune evasion (*lpsB*,* lpxABDLM*), acinetobactin (*barAB*,* bauA-F*, *basABDFGHIJ*,* entE*), quorum sensing regulation (*abaIR*), two-component system regulation (*bfmRS*), and serum resistance (*pbpG*) were detected in all the tested isolates. However, some genes were not found in all the isolates. ST85 (GC9) isolates were the only tested strains that harbored the *ugd3* gene (responsible for anti-phagocytic activity). On the other hand, *pilE* (responsible for adherence) and *hemO* (responsible for heme utilization) were not detected in these isolates (Fig. 1). Moreover, a gene coding for an oxidoreductase protein responsible for adherence was only detected in AB44-AUFP. According to VFDB, 2 and 9 additional open reading frames, with undetermined gene names, coding for heme utilization were present in ST85 and ST158, respectively (data not shown).

### Phenotypic characterization of antimicrobial resistance

**Fig. 2:**
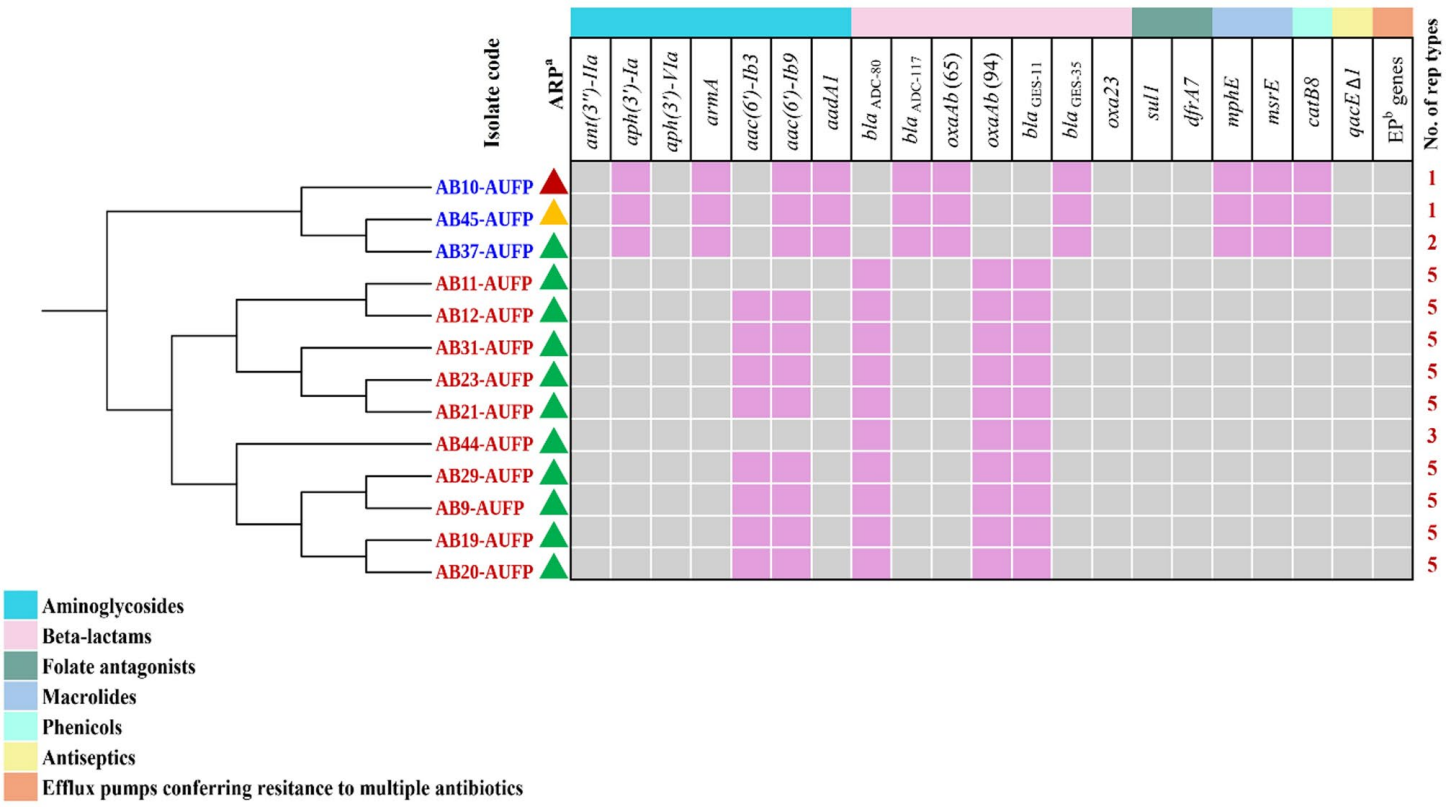
Illustration of the core gene phylogeny, antibiotic resistance phenotype and genotype, and the number of rep types for the 13 isolates. Blue labeling refers to ST85 (GC9) isolates, while red labels indicate the isolates that belong to ST158 (GC10). ^a^Antibiotic resistance pattern: the red triangle refers to the resistance to all the tested antibiotics except minocycline and colistin. The green triangle indicates the resistance to all the antibiotics under test except tetracycline, doxycycline, minocycline, and colistin. The orange triangle represents the resistance to all the tested antibiotics except tobramycin, gentamicin, tetracycline, doxycycline, minocycline, and colistin. Grey- and light orchid-colored squares in the heatmap represent the presence and absence of the resistance gene, respectively. ^b^Efflux pump genes: *abaF*,* abaQ*,* amvA*,* adeABFGHIJKLNRS*, and *abeMS*

The collected isolates were tested for their antimicrobial susceptibility to selected antibiotics using the Kirby-Bauer disk diffusion method or the VITEK^®^ 2 Compact system (in the case of colistin sensitivity detection). All the isolates were sensitive to colistin and minocycline. Moreover, all the isolates displayed the same resistance pattern except AB10-AUFP and AB45-AUFP (Fig. 2). It is noteworthy that some colonies were observed in the inhibition zones of doxycycline and minocycline antibiotics in the case of AB19-AUFP, AB20-AUFP, and AB21-AUFP isolates.

Upon the phenotypic detection of carbapenemase activity using THT, all isolates were positive for carbapenemase production manifested by clearly visible clover leaf-type indentation in the carbapenem disc inhibition zone at the site of intersection of the indicator strain, *E. coli* NCTC 10418, and the tested clinical isolate.

### Genomic analysis of the antimicrobial resistance determinants

The WGS analysis showed that the tested isolates harbored various antibiotic resistance genes that confer resistance to multiple classes of antibiotics. Genes coding for aminoglycoside resistance were remarkably abundant in the tested isolates. *ant(3’’)-IIa* and *aph(3’)-VIa* genes were detected in all the isolates, while, the genes *aph(3’)-Ia*,* armA*, and *aadA1* were exclusively present in all the isolates belonging to ST158 (GC10). Notably, *aph(3’)-VIa* gene was present in the long-read assembly of AB11-AUFP despite its absence in the short-read assembled contigs of the same isolate. Moreover, *aac(6’)-Ib3* gene was found in the short-read contigs of AB10-AUFP and AB45-AUFP as well as the long-read assemblies of AB11-AUFP, AB37-AUFP, and AB44-AUFP. Additionally, the *aac(6’)-Ib9* gene was only detected in the long-read assemblies of AB11-AUFP and AB44-AUFP (Fig. 2).

The isolates belonging to the 2 sequence types, ST85 (GC9) and ST158 (GC10), possessed different variants of beta-lactamase genes. The *bla*_ADC−80_, *oxaAb*(94), and *bla*_GES−11_ alleles were detected in the isolates related to ST85. On the other hand, isolates of ST158 harbored the *bla*_ADC−117_, *oxaAb*(65), and *bla*_GES−35_ alleles. The carbapenemase gene (*oxa23*) was found in all the tested isolates (Fig. 2).

Additionally, genes conferring resistance to sulfonamides *(sul1)* and trimethoprim (*dfrA7)* were present in all the isolates. However, macrolide (*mph(E)*,* msr(E)*), and chloramphenicol *(catB8)* resistance genes were only found in the isolates belonging to ST158 (GC10). The antiseptic resistance gene (*qacE∆1*), coding for an efflux pump, was detected in all the tested isolates. Moreover, numerous genes coding for different families of efflux pumps namely, *abaF*,* abaQ*,* amvA*, (MFS family), *adeABFGHIJKLNRS* (RND family), *abeS* (SMR family), and *abeM* (MATE family), that are responsible for multi-drug resistance were also found in all the tested isolates (Fig. 2).

#### Mutation analysis of QRDR

The mutation analysis of QRDR in *gyrA* and *parC* conferring fluoroquinolone resistance revealed the presence of multiple mutations as compared to *A. baumannii* ATCC 19,606. An S81L mutation in GyrA was detected in all the tested isolates. Moreover, other mutations were detected in GyrA including E85G, found in AB21-AUFP and AB23-AUFP isolates, as well as E151V which was detected in AB31-AUFP. Furthermore, mutations in GyrA were exclusively found in isolates of specific STs. The 3 isolates belonging to ST85 (GC9) harbored an E85V mutation, whereas A363T and G882S mutations were detected in isolates of ST158 (GC10). Similarly, regarding ParC mutations, E88K and G569S were found in isolates related to ST85, while isolates belonging to ST158 possessed S84L and S467G mutations.

#### Identification of rep types

**Fig. 3:**
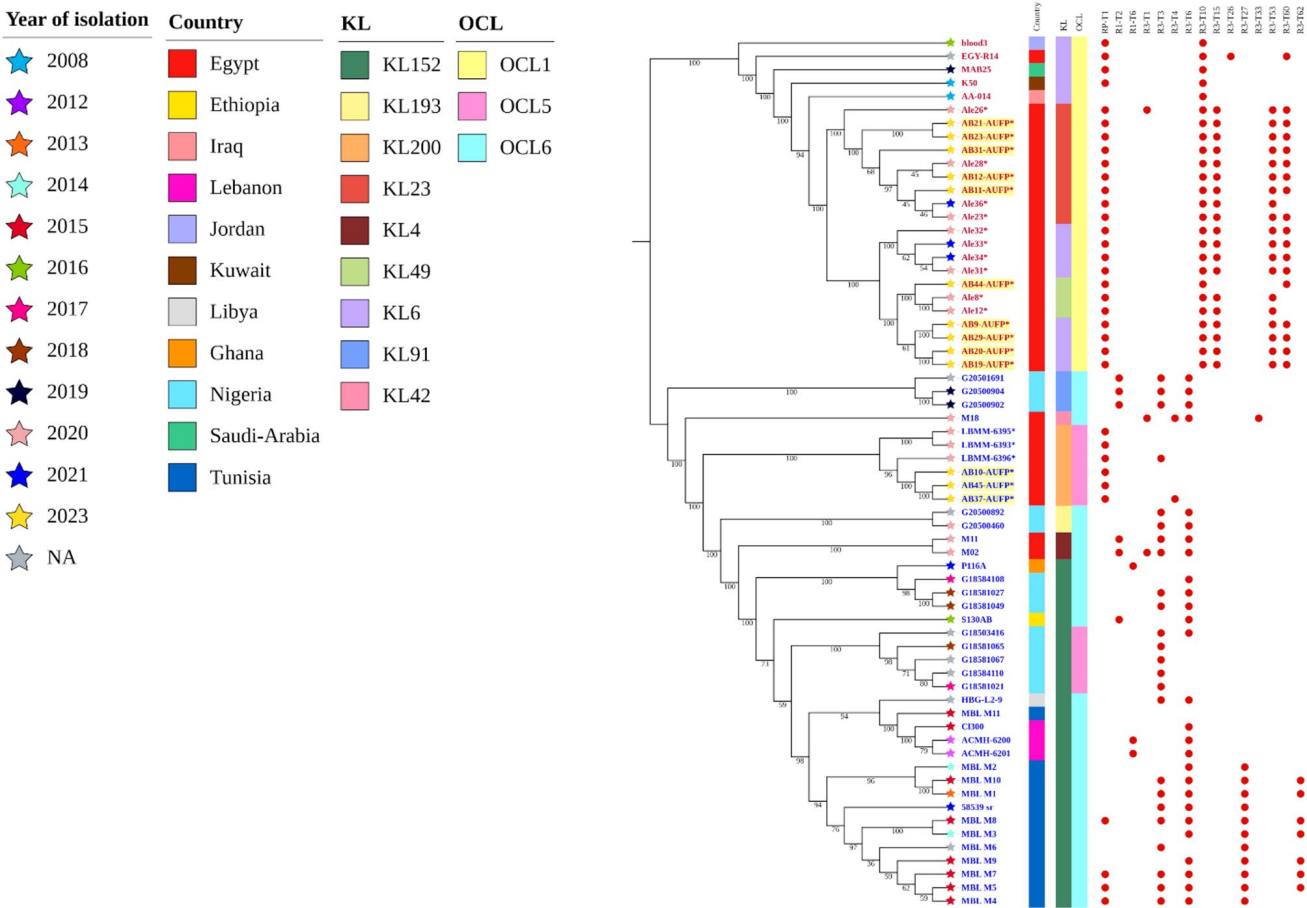
Core genome analysis using a midpoint-rooted maximum likelihood phylogenetic tree of 65 *A. baumannii* clinical isolates belonging to ST85 (GC9) or ST158 (GC10). Leaves of the tree are labeled with strain codes. The isolate codes in red represent the ST158 strains, while the blue labels indicate the isolates belonging to ST85. The labels highlighted in yellow indicate the isolates included in this study. *The labels with asterisks represent the strains collected from Alexandria, while the other Egyptian strains were obtained from Cairo. Branch labels indicate the level of support using 1000 bootstraps. The color strips (from left to right) show the country of isolation, KL, and OCL of the isolates. The distribution of rep types among the strains is displayed where the red circles indicate the presence of the corresponding rep type

The investigation of rep types using the *Acinetobacter Plasmid Typing database* showed the presence of 6 unique rep types belonging to R3 or RP rep families. Five rep variants, RP-T1, R3-T10, R3-T15, R3-T53, and R3-T60, were detected in all the isolates belonging to ST158 (GC10) except AB44-AUFP which only harbored the RP-T1, R3-T10, and R3-T60 rep types. RP-T1 was also detected in all the 3 isolates belonging to ST85 (GC9). Additionally, AB37-AUFP harbored R3-T4 (Figs. 2 and 3).

### Phylogenetic analysis

The core genome analysis using a maximum likelihood phylogenetic tree of 65 *A. baumannii* clinical isolates including the 13 isolates in this study is shown in Fig. 3. Two major clades were observed, corresponding to Institut Pasteur, ST85/GC9 (40 isolates) and ST158/GC10 (25 isolates). The isolates included in the phylogenetic analysis were collected at different time points between 2008 and 2023 and isolated from various geographical sources across the Middle East or Africa regions. It is noteworthy that the thirteen isolates in the present study were clustered together forming subclades along with other isolates from Alexandria/Egypt, but separately from the other Egyptian isolates from Cairo (Fig. 3) (Additional files 1 and 2).

Regarding ST85 (GC9) isolates, it was noticed that all the Egyptian isolates from Alexandria belonged to an ST85 lineage quite separate from the other ST85 isolates (demonstrated by a long branch length) **(**Additional file 1). For ST158 (GC10) isolates, it was notable that all the strains from Alexandria/Egypt were phylogenetically distinct from other countries where all strains were clustered in a single clade. Additionally, 6 ST158 lineages were circulating among the Alexandrian strains (Additional file 2).

#### Identification of KL and OCL types

It was noticed that only 3 KL types (KL6, KL23, and KL49) were recognized among ST158 (GC10) isolates, with KL6 being the most abundant (52%). On the other hand, KL152 was the predominant KL type (65%) among ST85 (GC9) isolates that were phylogenetically close to each other, while the rest of the isolates in this clade harbored other KL types namely KL193, KL200, KL4, KL91, and KL42. Regarding OCL typing, OCL1 was the only OCL type found among ST158 isolates whereas OCL6 and OCL5 were present in ca. 72 and ca. 27% of ST85 isolates, respectively (Fig. 3) (Additional files 1 and 2).

#### Analysis of rep types

Rep type analysis of the studied isolates revealed that RP-T1 and R3-T10 were the most abundant rep types (96% and 100%, respectively) among ST158 (GC10) isolates. Moreover, R3-T15, R3-T53, and R3-T60 were also widespread (76%, 76%, and 72%, respectively) among these isolates. On the other hand, R3-T3 and R3-T6 were present in ca. 60% and 68% of ST85 (GC9) isolates, respectively, while both rep types co-existed in ca. 45% of the isolates. The 6 ST85 isolates, from Alexandria/Egypt, as well as 4 ST85 isolates from Tunisia harbored RP-T1 (Fig. 3) (Additional files 1 and 2).

#### Investigation of antibiotic resistance genes

Carbapenemase genes, including *bla*_NDM−1_ and *oxa23*, have been reported to be endemically produced by MDRAB isolates, belonging to multiple clones, resulting in clinical outbreaks in North Africa and the Middle East [68]. Upon the genotypic analysis of the resistance determinants, depicted in Additional file 3, it was evident that most of the isolates (31 out of 40 isolates) belonging to ST85 (GC9) harbored the *bla*_NDM−1_ gene which produces the NDM-1 beta-lactamase, while 3 isolates carried *bla*_NDM−44_. The *bla*_NDM_ gene was absent in all 6 ST85 isolates originating in Alexandria/Egypt but was present in the isolates from Cairo. These 6 isolates formed a distinct clade within the ST85 phylogeny and carried the *oxa23* gene (Additional file 1). The *bla*_NDM_ and *oxa23* genes co-existed in 4 ST85 Tunisian isolates. Interestingly, these 6 Alexandrian isolates and 4 Tunisian isolates are the only ST85 isolates carrying the RP-T1 rep type and the *oxa23* gene, suggesting that this plasmid rep type might be the one carrying the *oxa23* gene. It was also noticed that 84% of ST158 (GC10) isolates, where the RP-T1 rep type was detected, harbored *oxa23* with *bla*_NDM_ being absent (Additional file 3) (Fig. 3).

The *bla*_GES_ genes are among the Extended-Spectrum β-Lactamase (ESBL) genes that have been frequently reported in the Mediterranean region and the Middle Eastern countries [69]. Two variants of the *bla*_GES_ gene were detected among the studied strains from both sequence types. The *bla*_GES−11_ gene was present in the 6 ST85 isolates from Alexandria/Egypt in addition to 3 strains belonging to ST158. On the other hand, the *bla*_GES−35_ gene was found in 68% of ST158 strains. Notably, RP-T1 rep type was detected in all the isolates with the *bla*_GES_ gene, suggesting that this gene might be carried on the RP-T1 plasmid as previously found [55] (Additional file 3) (Fig. 3).

It is noteworthy that the *sul1* and *qacE∆1* genes were also found to be present in all the isolates carrying RP-T1 rep type. On the other hand, *sul2* was commonly detected in all the ST85 strains except the 6 ST85 isolates from Alexandria/Egypt (Additional file 3) (Fig. 3).

Interestingly, five variants of the ADC protein were detected among ST85 isolates (3 assigned alleles, *bla*_ADC−80_, _−176_, _−258_, and 2 unassigned alleles that were not given a number in the NCBI database) where *bla*_ADC−80_ was the most prevalent *bla*_ADC_ variant (found in 17/40 isolates). Aside from a 15 bp duplication in *bla*_ADC−176_ and _− 258_ (resulting in a duplication of 5 amino acids), these five alleles differed by between 4 and 19 nucleotides from one another, representing an unusually high degree of variation in *bla*_ADC_ for a single ST. Four ST85 strains harbored two variants of the *bla*_ADC_ gene. The strain designated as P116A harbored 2 variants of *bla*_ADC_ gene, *bla*_ADC−80_ and another variant that showed 100% nucleotide identity with *bla*_ADC−176_, however, 4 nucleotides were missing at the beginning of the gene. The Cl300 strain carried an unassigned *bla*_ADC_ gene allele, showing 1 amino acid variation (V119E) from *bla*_ADC−80_. MBL_M11 also harbored an unassigned variant of the *bla*_ADC_ gene that differed from *bla*_ADC−80_ by four amino acids (R2Q, G24D, V119E, and T270A) (Additional file 3).

### Genomic analysis of plasmid structures

#### Characterization of pAB11-AUFP1, pAB44-AUFP1, and pAB37-AUFP1 plasmids

**Fig. 4:**
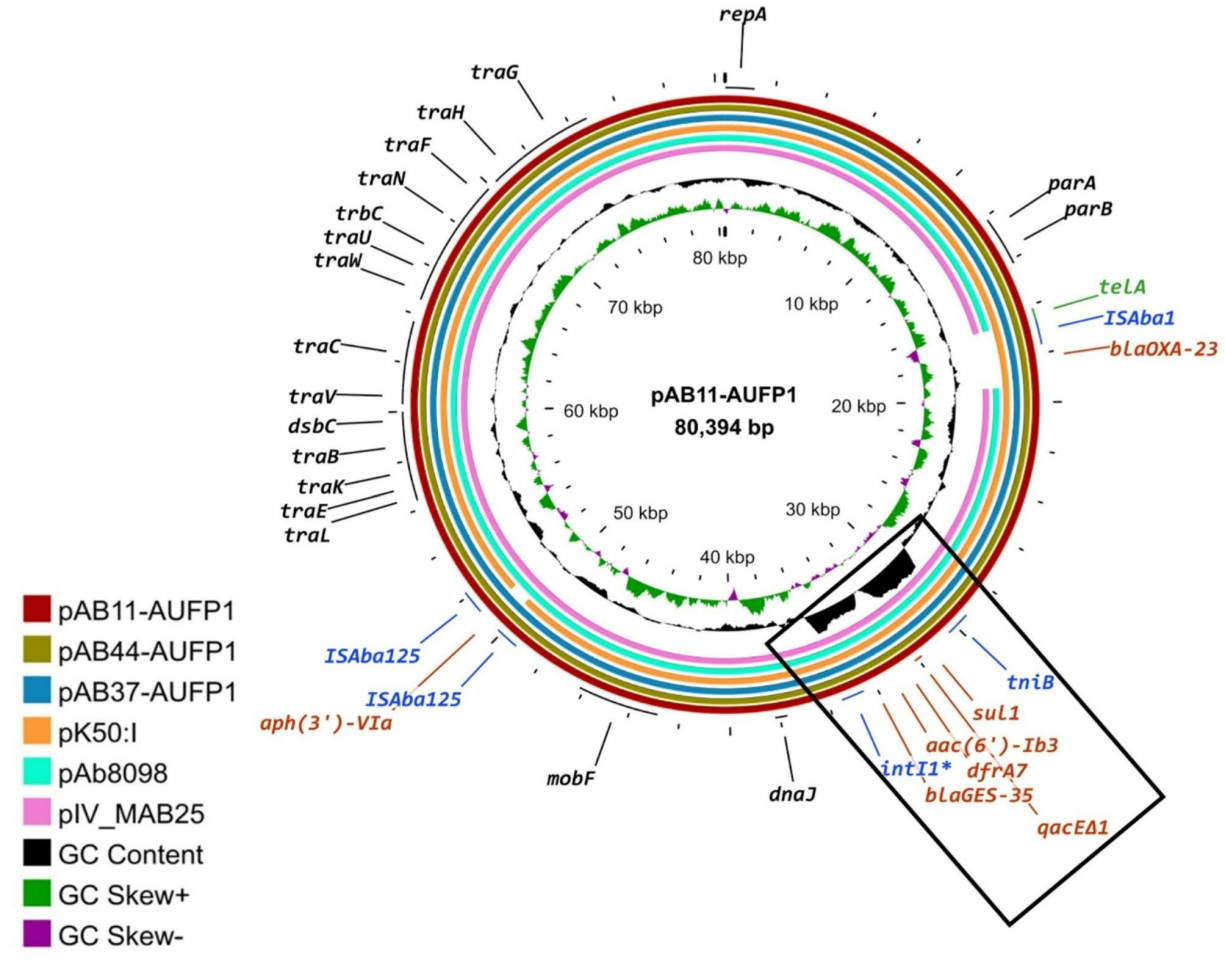
Comparison of RP-T1 plasmids from the clinical isolates AB11-AUFP1, AB44-AUFP1, and AB37-AUFP1 with similar plasmids obtained from the NCBI database. The labels in the outermost ring represent the gene annotation corresponding to AB11-AUFP1: antibiotic resistance (red), heavy metal resistance (green), mobile elements (blue), and plasmid replication, conjugation transfer, mobilization, stability, and segregation (black). The region marked by the black rectangle represents a putative resistance island, with a relatively high GC content, flanked by miniature inverted-repeat transposable elements (MITEs), comprising the resistance genes *sul1*,* aac(6’)-Ib3*,* dfrA7*,* qacE∆1*,* and bla*_GES_ that constitute an integron region. Regions of genomic homology, covered by BLASTn, are represented by a solid color whereas gaps refer to non-homologous regions. *Incomplete gene

Three plasmids, pAB11-AUFP1, pAB44-AUFP1, and pAB37-AUFP1, were recovered from long-read assemblies of the corresponding isolates using Plassembler. The plasmids, pAB11-AUFP1, pAB44-AUFP1, and pAB37-AUFP1, were 80,394, 80,395, and 80,390 bp long, respectively. Both pAB11-AUFP1 and pAB44-AUFP1 plasmids contained 103 CDS whereas 100 CDS were detected in pAB37-AUFP1. The three plasmids were likely conjugative, as predicted by Mobsuite, and had a GC content of ca. 35.5%. A high percentage of nucleotide identity, ranging between 99.98–100%, was detected upon the BLASTn analysis of the sequences of the three plasmids. They harbored genes that code for essential proteins including plasmid replication protein (coded by *repA*) that belongs to the rep type RP-T1, conjugative transfer and mobilization proteins (coded by *tra* and *mobF* genes), and stable maintenance proteins to ensure plasmid propagation, survival, and segregation (coded by *parA* and *parB* genes as well as genes coding for type II toxin–antitoxin system RelE/ParE family toxin). Moreover, a gene coding for a tellurite resistance protein (*telA*) together with mobile elements were found on the 3 plasmids. The transposon Tn*aphA6* was detected carrying the *aph(3’)-VIa* resistance gene flanked by 2 copies of IS*Aba125*. IS*Aba1* was also located directly upstream of *oxa23*, while an incomplete ATPase-encoding gene, ATPaseΔ, was downstream of *oxa23* composing Tn*2008*. The 3 plasmids harbored a class 1 integron incorporating *sul1*,* aac(6’)-Ib3*,* dfrA7*,* qacE∆1*,* and bla*_GES_ (with different variants) that is probably a part of a putative resistance island, predicted by IslandViewer4, with a relatively high GC content flanked by 439 bp miniature inverted-repeat transposable elements (MITEs) (Fig. 4).

Comparative genomic analysis of the 3 plasmids with closely related plasmids from Middle Eastern or African countries, belonging to ST158, using BLASTn revealed a high percentage identity (> 99%) and query coverage (≥ 99%) with pK50:I plasmid from Kuwait (accession no. LT984690.1) and pIV_MAB25 plasmid from Saudi Arabia (accession no. CP121592). Furthermore, there was a high sequence similarity between the 3 studied plasmids and the pAb8098 plasmid from Tunisia (accession no. KY022424.1) with about 94% query coverage and above 99% nucleotide identity (Fig. 4). Interestingly, it was noticed that the integron carried on our 3 plasmids was identical to the one present on plasmid pAb8098, however, pAb8098 harbored a different variant of *bla*_GES_ (*bla*_GES−14_) that is known for its carbapenemase activity. Moreover, an additional integron in pAb8098 containing the genes *strAB*, *aadB*, *aadA2*, and *cmlA5* was fused forming a complex class 1 integron, flanked by MITEs, that was missing on our studied plasmids.

It was noticed that our plasmids harbored some important features that were missing in the other plasmids. As inferred from the phylogenetic analyses above, these RP-T1 plasmids carried IS*Aba1* and *oxa23*, which were absent in pIV_MAB25 and pAb8098 plasmids (Fig. 4). However, in the MAB25 isolate, harboring pIV_MAB25 plasmid, *oxa23* was located on the chromosome; therefore, in some instances, the movement of *oxa23* between RP-T1 plasmids and the chromosome may be occurring. Additionally, the transposon Tn*aphA6* was not detected in the pK50:I plasmid.

#### Characterization of other plasmids in the selected isolates

Small plasmids (pAB11-AUFP2 and pAB44-AUFP2) of 8,251 bp were detected in the corresponding isolates. They carried a replication initiation protein that belonged to rep type R3-T10 and they also harbored genes encoding some virulence factors such as septicolysin and TonB-dependent receptors. Moreover, AB37-AUFP contained an 11,294 bp plasmid (pAB37-AUFP2) with a replication initiation protein belonging to rep type R3-T4, and it carried the same virulence genes as pAB11-AUFP2 and pAB44-AUFP2 together with other IS elements whose sequences were closely similar to the IS*Aba32* group. A third small plasmid, pAB11-AUFP3, of 2277 bp was present in AB11-AUFP that carried a replication initiation protein belonging to rep type R3-T15 together with an incomplete replicase gene related to the rep type R3-T53.

### Comparative chromosomal analysis of AB11-AUFP, AB44-AUFP, and AB37-AUFP

**Fig. 5:**
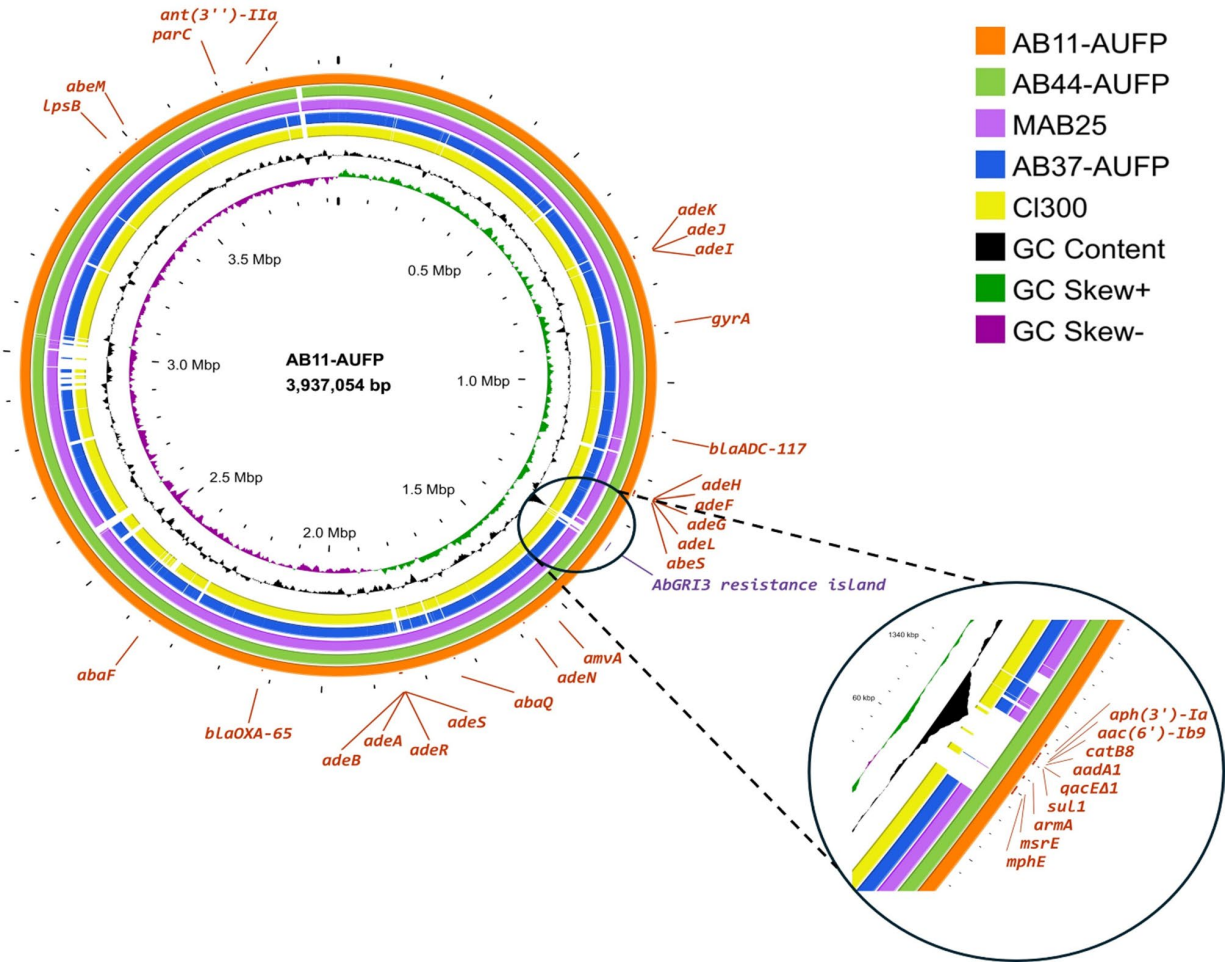
Genomic comparison of chromosomes of the clinical isolates AB11-AUFP, AB44-AUFP, and AB37-AUFP together with similar complete chromosomes retrieved from the NCBI database for *A. baumannii* strains using Proksee. The labels in the outermost ring represent the gene annotation related to resistance genes (red) corresponding to AB11-AUFP as predicted by the CARD resistance gene identifier tool offered by Proksee. A magnified view of the AbGRI3 resistance island (purple label) with a relatively high GC content showing 9 genes that confer resistance to various antimicrobial classes. Regions of genomic homology, covered by BLASTn, are represented by a solid color whereas gaps refer to non-homologous regions

The three complete chromosomes of AB11-AUFP, AB44-AUFP, and AB37-AUFP were recovered from long-read assemblies using the Hybracter pipeline with chromosome lengths 3,937,054, 3,964,414, and 4,008,721 bp, respectively. Figure 5 illustrates the comparative chromosomal analysis of these isolates together with other closely related complete chromosomes deposited in the NCBI database from countries in the Middle East using the chromosome of AB11-AUFP as the backbone. The BLASTn analysis of the chromosomes of AB11-AUFP and AB44-AUFP isolates, belonging to ST158 (GC10), showed high sequence similarity that reached about 99.97% (98% sequence length). Furthermore, there was a 99.97% nucleotide identity (97% query coverage) between the complete chromosome of the MAB25 isolate from Saudi Arabia (accession no. CP121591.1; ST158) and AB11-AUFP. Additionally, the AB37-AUFP (ST85) chromosome displayed a high sequence similarity with AB11-AUFP (ca. 98%) but with a lower percentage query coverage (89%) illustrated by white gaps in Fig. 5. On the other hand, AB37-AUFP showed a high sequence similarity and query coverage (99.97% and 94%, respectively) with Cl300 isolate from Lebanon (accession no. CP082952.1; ST85). It was noticed that all the chromosomes shared almost the same chromosomally encoded AMR genes. However, the AbGRI3 resistance island, predicted by IslandViewer4, with a relatively high GC content, was only detected on the AB11-AUFP and AB44-AUFP chromosomes. This resistance island comprised 9 genes that confer resistance to various antimicrobial classes (Fig. 5).

#### Genetic configuration of AbGRI3 resistance island and its comparison to closely related islands

**Fig. 6:**
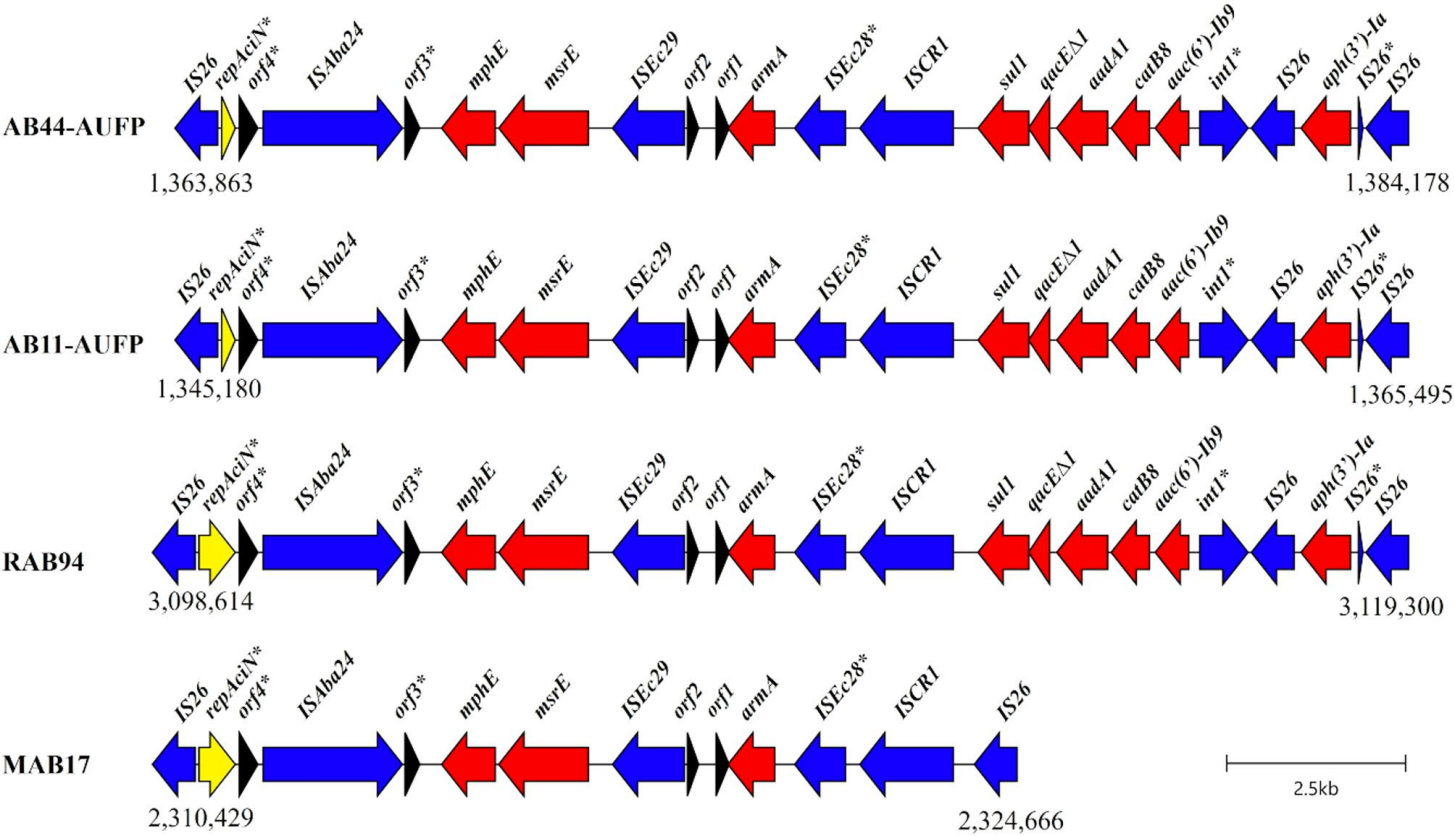
Schematic representation of AbGRI3-2 resistance island found on AB11-AUFP and AB44-AUFP chromosomes compared to variants of the island that are on the complete chromosomes of *A. baumannii* strains recovered from Saudi Arabia (RAB94 and MAB17). Red, blue, and yellow arrows represent open reading frames corresponding to resistance genes, mobile elements, and replicase genes, respectively. Black arrows refer to miscellaneous open reading frames; orf1: aminomethyltransferase beta-barrel domain-containing protein, orf2: hypothetical protein, orf3: DNA-binding protein, and orf4: plasmid replication DNA-binding protein. ORFs are drawn to scale. *Incomplete gene. The figure was created using Clinker software

An IS*26*-flanked chromosomal RI of 20,316 bp was detected, by IslandViewer4, on AB11-AUFP and AB44-AUFP chromosomes. It was previously identified as AbGRI3-2 which included both Tn*6180* and Tn*6179* [70]. Tn*6180* was formerly characterized by the presence of IS*Aba24* and was found to harbor *mphE*,* msrE*, and *armA* resistance genes together with a class 1 integron carrying an incomplete integrase, and a gene cassette that included *sul1*,* qacE∆1*, *aadA1*,* catB8*, and *aac(6’)-Ib9* genes. Tn*6179* was found adjacent to, and sharing an IS*26*, with Tn*6180*. It contained the *aph(3’)-Ia* gene, bound by two IS*26* elements. The AbGRI3-2 island carried an incomplete replicase gene (*repAciN*), belonging to the rep type R3-T60, that has been previously shown to be a nonfunctional replication initiation protein. Hence, it was concluded that it is unlikely that this structure could exist as a stable plasmid [70] (Fig. 6).

The structure of AbGRI3-2 RI was identical on both AB11-AUFP and AB44-AUFP chromosomes. The structure of the island in our isolates was compared to closely related islands on complete chromosomes of isolates from Middle Eastern countries collected from Saudi Arabia, using BLASTn analysis and the Clinker tool. It was found that the chromosome of RAB94 isolate (accession no. CP121563.1; ST570/GC2) carried almost the same AbGRI3-2 island as in our isolates with 99.9% homology. On the other hand, the chromosome of MAB17 isolate (accession no. CP121595.1; ST570/GC2) carried another variant of AbGRI3 that had a sequence similarity of 100% but with only 77% of the sequence length of the discovered island in our isolates. This shorter variant of the island was of 14,238 bp length and was considered an IS*26*-bound RI; however, it lacked the integron region of Tn*6180* together with the complete absence of Tn*6179* that was present in AbGRI3-2 (Fig. 6).

### Analysis of the draft genomes for mobile element characterization in the short-read assemblies of the isolates

The analysis of the draft genomes of the remaining 10 isolates revealed that RP-T1-carrying contigs probably belonged to putative conjugative plasmids. In contrast, the rest of the predicted plasmids were non-mobilizable as postulated by the Mobsuite tool. Interestingly, *oxa23* existed on the same short-read contig carrying the RP-T1 rep type in all the isolates, except AB9-AUFP, AB19-AUFP, AB29-AUFP, and AB31-AUFP, which suggests the presence of this gene on a putative conjugative plasmid in these isolates. Additionally, all the resistance genes found on the previously characterized conjugative plasmid from the long-read assemblies were also identified in the short-read data of the rest of the isolates which might infer that all the studied isolates carried this plasmid. However, this could not be confirmed as these resistance genes were carried on multiple short-read contigs in each sample. Moreover, *mphE*,* msrE*, and *armA* resistance genes co-existed on the same short-read contig carrying the R3-T60 rep type in all the tested ST158 isolates suggesting that the putative resistance island AbGRI3 might be found in these isolates. The rest of the resistance genes found on the putative chromosomal island AbGRI3-2 were also identified in the short-read data of all the isolates belonging to ST158 (GC10), but their location could not be confirmed.

### The combined activity of repurposed drugs and antibiotics

**Fig. 7:**
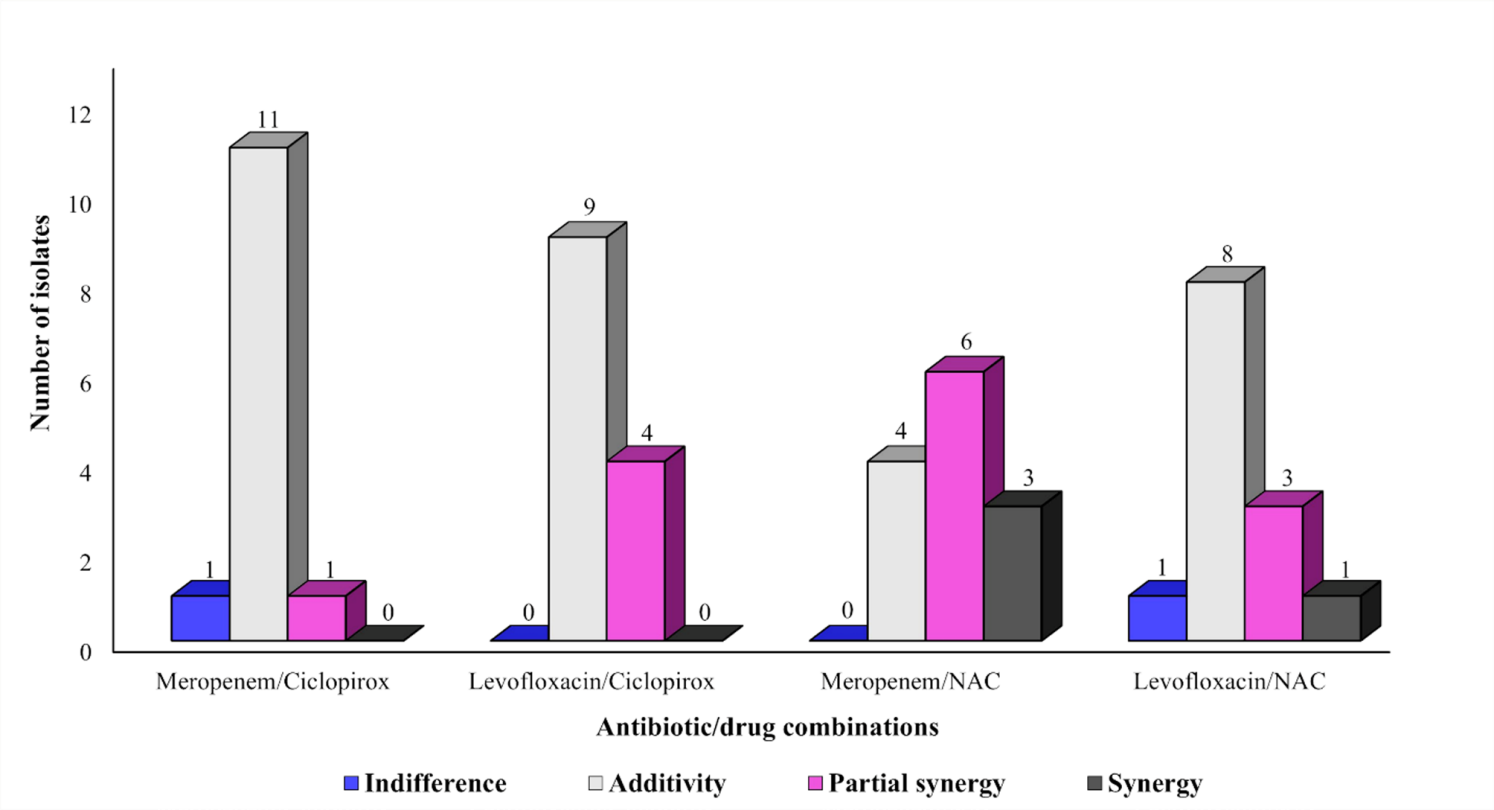
The efficacy of meropenem or levofloxacin antibiotics when combined with each ciclopirox or NAC, as repurposed drugs, against 13 *A. baumannii* clinical isolates using the checkerboard assay

The efficacy of combining meropenem or levofloxacin antibiotics with each of ciclopirox or NAC, as repurposed drugs, against 13 *A. baumannii* clinical isolates was determined using the checkerboard assay. The minimum inhibitory concentration ranges for meropenem, levofloxacin, ciclopirox, and NAC were between 32–128 μg/mL, 16–64 µg/mL, 4.875–9.75 µg/mL, and 3.125–6.25 mg/mL, respectively. Synergism was evident in the case of the meropenem/NAC combination against 3 isolates, AB12-AUFP, AB31-AUFP, and AB37-AUFP with a fold reduction in the MIC of meropenem ranging between 4 and 16-folds at ½ MIC of NAC. The levofloxacin/NAC combination also displayed a synergistic action against only one isolate (AB11-AUFP) with a fourfold reduction in the MIC of levofloxacin at ½ MIC of NAC. Moreover, partial synergism was noticeable in the case of the meropenem/NAC combination (6/13 isolates) followed by the levofloxacin/ciclopirox, levofloxacin/NAC, and meropenem/ciclopirox combinations (4/13, 3/13, and 1/13 isolate(s), respectively). The 4 combinations displayed an additive action against some of the tested isolates with the highest additive effect recorded in the case of meropenem/ciclopirox (11/13 isolates), whereas the meropenem/NAC combination showed the least additivity against only 4 isolates. Indifference was observed in 2 combinations; meropenem/ciclopirox and levofloxacin/NAC combinations against only 1 isolate for each. None of the evaluated combinations showed antagonistic action against the tested isolates (Fig. 7).

## Discussion

MDRAB clinical isolates have a diverse global geographical distribution. MDRAB rates have been reported to range between 77 and 87% in Latin America, Asia, and Africa, and 47% in North America, whereas they exceeded 93% in the Middle East and Europe [1]. This study adds to the previously published research on the widespread distribution and potential endemic nature of certain STs throughout North Africa and the Middle East.

The present work characterized the resistance and virulence profiles of MDRAB, belonging to the recently emerged sequence types, ST85 (GC9) and ST158 (GC10), collected from Alexandria, Egypt. Thirteen *A. baumannii* isolates were identified where 3 and 10 clinical isolates belonged to ST85 (GC9) and ST158 (GC10), respectively. GC9 (clonal complex 464) is considered among the youngest global clones that have been introduced since 2019 [17, 71]. It is a high-risk global clone that has been widely reported in the Middle Eastern and African regions [15, 72, 73]. Similarly, ST158 (GC10) strains have been previously recognized to represent a region-specific lineage as they were exclusively found in countries including those in the Middle East, North Africa, and the Mediterranean region [8, 74, 75]. The presence of *A. baumannii* isolates belonging to ST85 and ST158 in Egypt has been previously reported [11, 69, 72, 75].

*A. baumannii* is associated with a wide range of nosocomial infections. MDRAB is a predominant cause of respiratory infections in ICU patients and is one of the most challenging nosocomial pathogens to manage [76]. *A. baumannii* frequently appears in sputum cultures of patients with prolonged hospital stays and is a common cause of hospital-acquired pneumonia, typically linked to poor outcomes and high mortality rates [77]. Multiple virulence phenotypes contribute to lung infections caused by *A. baumannii*, such as biofilm formation, adherence to biotic surfaces, invasion of host cells, induction of apoptosis, resistance to oxidative stress, and iron regulation [78]. In the current study, 10/13 isolates were collected from respiratory clinical specimens. They were associated with several virulence factors, namely biofilm formation, twitching motility, phospholipase production, and protease activity. Under the tested experimental conditions, ST85 isolates showed moderate biofilm formation, however, one isolate was a strong biofilm former. On the other hand, all ST158 isolates displayed weak biofilm formation except 2 isolates which were moderate biofilm formers. In a previous study of MDRAB clinical isolates belonging to multiple STs from Alexandria, Egypt, it was noticed that isolates belonging to ST158 showed a biofilm formation ability that was markedly weaker than those belonging to ST85 and other STs [69]. The correlation between biofilm formation and antibiotic resistance has been controversial. Qi et al. reported a negative correlation between antibiotic resistance and biofilm formation capacity in *A. baumannii* strains, where MDRAB strains tended to produce weaker biofilms than non-MDR strains [79]. In contrast, previous studies confirmed the potential strong correlation between antibiotic resistance and biofilm formation where biofilms act as barriers against antibiotic diffusion to the bacterial cells and help *A. baumannii* strains develop antibiotic resistance [80, 81]. Positive twitching motility was evident in all the tested isolates except the ST85 isolate (AB45-AUFP). It is noteworthy that AB45-AUFP, which showed strong biofilm formation, was a negative twitcher. However, the 8 ST158 isolates which were weak biofilm formers showed positive twitching motility. The correlation between these two virulence factors in *A. baumannii* has been debatable. It was previously reported that twitching motility, mediated by type IV pili, is involved in biofilm formation [82, 83] and was found to be a common trait in abundant biofilm formers [35]. In contrast, another study found no correlation between the twitching motility and biofilm formation or antibiotic resistance among the studied isolates [80]. Vijayakumar et al. conducted a study comparing the extent of biofilm formation and motility in MDRAB clinical isolates collected from blood and sputum samples. They found that respiratory isolates produced more biofilm and were less motile. They attributed this finding to many postulations including the production of reactive oxygen species in oxygen-rich environments, such as in the lungs, which might suppress the production of many factors, such as 1,3-diaminopropane, required for motility [34]. Other studies suggested that biofilm formation in *A. baumannii* might downregulate the genes responsible for motility as in other bacteria [34, 84, 85]. This would be consistent with our phenotypic observations for AB45-AUFP (isolated from MiniBAL) but not the case with the rest of our isolates obtained from respiratory samples as they displayed wide twitching zones combined with low or moderate biofilm-forming ability. Vijayakumar et al. also found that *A. baumannii* isolates from blood samples displayed relatively higher motility with less biofilm formation capacity [34]. In our work, the ST85 isolate AB37-AUFP, isolated from blood, showed the highest twitching motility and moderate biofilm formation capacity. Additionally, all but one isolate showed moderate phospholipase activity and all had strong protease activity. Phospholipase activity, mediated by phospholipase C and D enzymes, is an important factor in the pathogenicity of *A. baumannii*. This activity facilitates host cell lysis and is associated with serum resistance and epithelial cell invasion [86–88]. F. Naeimi Mazraeh et al. reported that protease activity, another important virulence factor in *A. baumannii* infections, was detected in ca. 45% of their tested isolates and they stated that this virulence phenotype was significantly correlated to the presence of the *pld* gene encoding phospholipase D [89]. Another study reported the ability of *A. baumannii* isolates to secrete proteases such as CpaA which contributes to the pathogen’s virulence by dysregulation of the host coagulation system [90].

In this study, the virulome analysis revealed that isolates of both ST85 and ST158 exhibit an almost comparable collection of virulence determinants. However, the *pilE* and *hemO* genes were not found in the isolates belonging to ST85 (GC9). The *pilE* gene plays a role in host cell adhesion [91] and is necessary for natural competence [92] as well as twitching motility [82]. Nonetheless, two ST85 isolates, AB10-AUFP and AB37-AUFP, displayed substantial twitching motility indicating that twitching motility is not solely mediated by *pilE*. *hemO* encodes for the “haem oxygenase” enzyme responsible for haem degradation and iron release. It is a part of the hemO gene cluster crucial for bacterial pathogenicity and dissemination systemically [93]. Here, we didn’t phenotypically investigate the impact of the absence of *hemO* on the haem uptake and utilization among ST85 isolates. However, its absence among ST85 isolates might reveal a difference in the iron acquisition strategies adopted by ST85 and ST158 isolates.

In Egypt, the current antibiotic resistance scenario is dreadful due to rapidly evolving MDR isolates in hospital settings. This is caused by the imprudent use of antibiotics and increased antibiotic pressure [12]. In the present study, all the isolates showed resistance to multiple classes of antibiotics namely beta-lactams (with carbapenemase production), aminoglycosides, fluoroquinolones, and sulfonamides. However, the ST85 isolate AB45-AUFP was sensitive to tobramycin and gentamicin. On the other hand, all isolates displayed susceptibility to colistin and minocycline, and 12/13 isolates were sensitive to tetracycline and doxycycline. GC9 (ST85) isolates were previously reported to show high resistance rates to carbapenems and other antimicrobials including aminoglycosides, and fluoroquinolones, while a low rate of resistance was observed against tetracyclines and colistin [73, 94]. Similarly, Sanchez-Urtaza et al. previously noticed reduced susceptibility among GC10 (ST158) isolates to carbapenems, aminoglycosides, fluoroquinolones, and sulfonamides whereas most of the isolates were sensitive to minocycline [69]. In contrast to our findings, resistance to colistin was previously reported in an ST158 isolate, in addition to other isolates belonging to the most widely disseminated GC2, which raises an alarming concern as colistin is used as a last resort treatment option against MDRAB [74]. Notably, the tested isolates of both sequence types harbored multiple antibiotic resistance determinants that were chromosomal- or plasmid-mediated. These data confirm that the remaining treatment options for infections caused by GC9 and GC10 isolates, in Egypt, are diminishing.

It is noteworthy that some colonies were observed in the inhibition zones of tested tetracycline antibiotics, doxycycline and minocycline, in the case of AB19-AUFP, AB20-AUFP, and AB21-AUFP isolates. Previous studies have noted the prevalence of heteroresistance in *A. baumannii* clinical isolates treated with tetracycline antibiotics, such as tigecycline and eravacycline. Enhanced expression of efflux pumps, including AdeABC and AdeRS, represents one of the major mechanisms contributing to the development of heteroresistance against such antibiotics [95, 96].

Genotypic analysis of fluoroquinolone resistance revealed that the S81L mutation in GyrA was highly conserved in all isolates. Moreover, ST-specific mutations in the QRDRs of GyrA or ParC were found. S84L mutation in ParC was detected in all ST158 (GC10) isolates, whereas E85V in GyrA and E88K in ParC were noticed in ST85 isolates (GC9). Similarly, Hamed et al. [11] reported the presence of the S81L amino acid alteration in GyrA in all the studied isolates belonging to multiple global clones (GC2, GC4, GC5, GC7, and GC9). Moreover, in their study, most of the isolates exhibited a GyrA^S81L^/ParC^S84L^ mutation pattern which was reported to be associated with increased resistance to fluoroquinolones in *A. baumannii* [11, 97, 98]. In addition, Hamed et al. noticed other mutations that might contribute to fluoroquinolone resistance such as E88K in ParC together with S81L and E85V in GyrA [11]. In our study, the genotypic analysis of fluoroquinolone resistance revealed the presence of other mutations which, to our knowledge, weren’t previously reported to be associated with fluoroquinolone resistance. These mutations included E85G, E151V, A636T, and G882S mutations in GyrA. The latter 2 mutations were exclusively detected in ST158. Moreover, S467G (ST158-specific) and G569S (ST85-specific) were found in ParC. In addition to mutations in the QRDRs of GyrA or ParC, fluoroquinolones are expelled by various efflux pumps encoded by genes, such as *adeABFGHIJK* (RND family); *abeS* (SMR family); and *abeM* (MATE family) [11]. These efflux pump encoding genes were detected in all our isolates.

In *A. baumannii*, carbapenemases, especially class D oxacillinases, are the main mediators of carbapenem resistance [94] where the carbapenemase *oxa23* is the most prevalent acquired oxacillinase in *A. baumannii* [94, 99]. In the current work, the *oxa23* gene was detected in 22/25 ST158 isolates and 10/40 ST85 isolates including our 13 isolates. Egypt is considered a hotspot for the prevalence of *oxa23* among multiple global clones including GC9 (ST85) and GC10 (ST158) [69, 75]. Class B metallo-β-lactamase genes, such as *bla*_NDM_, encode for carbapenemases that are endemically produced by MDRAB isolates in North Africa and the Middle East [68]. It was evident from the phylogenetic analysis that the *bla*_NDM−1_ gene was the most prevalent metallo-β-lactamase gene among ST85 isolates collected from various countries. The *bla*_NDM−1_ gene is frequently chromosomally located within a truncated Tn*125* [73, 100]. Notably, GC9 isolates might be considered a source of disseminating *bla*_NDM−1_ to other *A. baumannii* global clones or even among various bacterial species.

*A. baumannii* plasmids have been previously classified into 3 major rep types R1, R3, and RP type families. Plasmids belonging to R3 and RP families have been considered important carriers of antibiotic resistance determinants including those encoding resistance to carbapenems and aminoglycosides. They are among the major MGEs that cause the horizontal transfer of AMR genes, hence, allowing the spread of MDRAB strains and exaggerating the AMR problem [55]. Our phylogenetic analysis revealed that the RP-T1 rep type (also referred to as *repAci6*) was abundant in 24/25 ST158 isolates and 10 ST85 isolates. RP-T1 plasmids are among the highly disseminated plasmids between the major globally distributed clones of *A. baumannii*. They usually harbor the *oxa23* gene with or without the *aphA6* gene in addition to other resistance genes including *sul1*,* dfrA7*,* strAB*,* aacA4*,* aadA2*,* aadB*, *bla*_GES−11_, *cmlA1*, and *oxa58* [55]. Seven and six different R3 rep types were detected among the strains belonging to ST85 and ST158, respectively. It was previously found that the R3 family of plasmids is highly abundant and contains the most diverse variants compared to other rep types. Notably, not all R3 plasmid variants were reported to carry AMR genes [55]. The R1 rep type was the least detected and was only recognized in ST85 isolates (8/40 isolates). R1 plasmids have been considered less prevalent and generally small in size (2–3 kb). They do not commonly carry antibiotic resistance determinants hence they are not thought to contribute to the spread of antibiotic resistance [55].

The plasmids identified in our study closely resembled those detected in other ST158 strains circulating in the Middle East and North Africa including Kuwait [101], Saudi Arabia (accession no. CP121592), and Tunisia [102]. Interestingly, in a previous study, ST85 and ST158 Egyptian MDRAB strains harbored RP-T1 plasmids carrying antibiotic-resistance genes comparable to our plasmids [103]. A closely related plasmid was also detected in an MDRAB strain, from the United States, belonging to a widely disseminated high-risk global clone (GC1) [104]. Additionally, the plasmid pABUH, carrying the RP-T1 rep type, was previously detected in *A. baumannii* isolates belonging to GC2 (collected in the USA), this plasmid closely resembled our RP-T1 plasmids but lacked the integron region (90% query coverage, 100% percentage identity) [105]. Hence, we can conclude that RP-T1 plasmids propagate globally among multiple global clones, including the recently emerged GC9 and GC10.

The carbapenemase gene *oxa23* is usually accompanied by insertion elements and transposons, such as Tn*2006* and Tn*2008*, enabling its efficient spread on conjugative plasmids across various *A. baumannii* lineages [94, 99]. Tn*2008*, comprised of IS*Aba1*, *oxa23*, and ATPaseΔ, was carried on our conjugative RP-T1 plasmids. Similarly, *oxa23-*containing transposons, such as Tn*2008*, were previously detected on RP-T1 plasmids in *A. baumannii* clinical isolates belonging to multiple GCs including GC2 (the most prevalent high-risk global clone), GC9, and GC10 [101, 103, 105]. This confirms that RP-T1-associated conjugative plasmids are one of the major mobile elements facilitating the propagation of the *oxa23* gene among various global clones of *A. baumannii* thus exacerbating the dissemination of carbapenem resistance.

MITEs were reported to act as movable vectors for the spread of integrons [106]. A class 1 integron incorporating *sul1*,* aac(6’)-Ib3*,* dfrA7*,* qacE∆1*,* and bla*_GES_ (with different variants) was detected in our plasmids flanked by 439 bp MITEs. A similar integron has been previously found on a conjugative plasmid from Kuwait [101]. These 5 resistance determinants were also formerly recognized to be part of an incomplete class 1 integron surrounded by MITEs, provisionally recognized as MITE*Ab-IC10*, in ST158 isolates collected in the Middle East [8]. An expanded version of MITE*Ab-IC10* forming a complex class 1 integron, flanked by MITEs was also detected in plasmids carried by GC10 and GC1 isolates [2, 8, 102, 104]. It has been previously suggested that the high GC content of the integron region might constitute a putative resistance island of a non-*Acinetobacter* origin that could be mobilized by MITEs via direct transposition or homologous recombination. This might further enhance the dissemination of resistance genes to other bacteria [102, 104].

The co-existence of the mobile elements, Tn*2008*, Tn*aphA6*, and MITE*Ab-IC10*, on conjugative plasmids belonging to RP-T1, was previously detected in many ST158 isolates collected from the Middle East [8]. This indicates that GC10 (ST158) isolates are considered major sources of the spread of genes conferring resistance to clinically important antibiotics used to combat *A. baumannii* isolates.

In our study, small plasmids carrying virulence genes encoding septicolysin and TonB-dependent receptors were detected in AB11-AUFP, AB44-AUFP, and AB37-AUFP isolates. These plasmids belonged to R3-T10 or R3-T4 rep types. Septicolysin is considered a pore-forming toxin [107], whereas TonB-dependent receptor protein is potentially involved in iron acquisition and pathogenicity [108]. Similarly, these virulence determinants were previously recognized on plasmids of small size belonging to the Rep-3 family [101, 103]. This might highlight the crucial role of this family of plasmids in the dissemination of virulence determinants among *A. baumannii* isolates.

Resistance islands incorporate mobile elements such as transposons and integrons carrying various resistance genes [13]. AbGRI3 is a resistance island that was formerly characterized to encompass Tn*6180* which carried several resistance genes including *armA*,* msrE*, and *mphE* with or without a class 1 integron. In addition, Tn*6179* might be integrated into the RI carrying IS*26*-bracketed *aph(3’)-Ia* gene [70]. Different variants of AbGRI3 were detected in *A. baumannii* belonging to GC2 worldwide [13, 70, 109]. In our study, AB11-AUFP and AB44-AUFP isolates harbored the AbGRI3-2 RI on their chromosome. To our knowledge, this is the first study reporting the presence of AbGRI3 RI in *A. baumannii* strains belonging to ST158.

Repurposing off-patent medications that have already received FDA approval for other purposes can result in the emergence of novel antibacterial drugs [110]. This might be a promising alternative to de novo medication discovery and development that could aid in combating MDRAB [111]. Combining current medications with antibacterial agents that are implemented in clinical practice is also a valid option that has been extensively investigated [112].

NAC is a thoroughly studied medication with well-established safety. It is a commonly used drug with an excellent tolerability profile that primarily acts as a mucolytic agent and has anti-inflammatory and antioxidant activities. NAC was reported to exhibit antibacterial properties and enhance the effects of antibiotics in combating bacterial infections including those caused by MDR pathogens [23, 113]. The antibacterial effect of NAC might be attributed to the functional group -SH, which could break the S–S disulfide bridges in proteins present in the bacterial cells, leading to their denaturation and eventually inactivating their function. Moreover, NAC might inactivate several bacterial beta-lactamases including carbapenemases due to protein misfolding and subsequently loss of their function [23]. In our study, synergism was evident when combining NAC with each of meropenem and levofloxacin against 3 and 1 isolate(s), respectively. De Angelis et al. noticed a synergistic action of the meropenem/NAC combination against all the tested CRAB isolates [23]. However, the results obtained due to the co-administration of NAC with beta-lactams have been controversial [23, 114]. The efficacy of the levofloxacin/NAC combination against bacterial pathogens has also been debatable. NAC has been found to reduce the antibacterial activity of fluoroquinolones against bacterial strains such as *Escherichia coli*, *Klebsiella aerogenes*, and *Pseudomonas aeruginosa* [115]. In contrast, Landini et al. reported that the administration of NAC did not interfere with the activity of fluoroquinolones against the respiratory pathogens tested [116]. Moreover, Lea et al. noticed that the co-administration of NAC with ciprofloxacin inhibited the growth of *P. aeruginosa* in the planktonic and biofilm states [117]. Based on our findings, NAC did not hinder the action of levofloxacin against MDRAB isolates. NAC might enhance the antibacterial action of levofloxacin as synergism and partial synergism actions were noticed against 1 and 3 MDRAB isolates, respectively.

Ciclopirox is an excellent repurposed agent with a remarkable safety profile. It could inhibit the growth of MDR pathogens such as *A. baumannii*, *E. coli*, and *K. pneumoniae* clinical isolates [118]. Strikingly, in our study, ciclopirox exerted significant antibacterial activity against the tested MDRAB isolates with MIC values ranging between 4.875 and 9.75 µg/mL. Kimberly et al. reported a comparable MIC range (5–7 µg/mL) of ciclopirox against MDRAB strains [118]. In their study, they attributed the antibacterial activity of ciclopirox to iron chelation mechanisms in addition to other iron-independent pathways including altering the composition of bacterial lipopolysaccharide and affecting galactose metabolism. Unfortunately, ciclopirox did not exert any synergistic action when combined with meropenem or levofloxacin against MDRAB isolates using the in vitro checkerboard assay. However, partial synergism was noticeable in the case of the levofloxacin/ciclopirox and meropenem/ciclopirox combinations against 4 and 1 isolate(s), respectively.

In conclusion, this study provides further insights into the resistance and virulence profiles of the collected MDRAB isolates belonging to the recent global clones GC9 and GC10 circulating in the Middle East and North Africa. It demonstrates that these emerging global clones are common and endemic in the region, and this subsequently necessitates strategies to manage future infections and curb ongoing MDRAB outbreaks in low and middle-income countries such as Egypt. Repurposing NAC as an adjuvant with antibiotics such as meropenem and levofloxacin might be promising to combat MDRAB isolates belonging to recent lineages. However, it is important to acknowledge certain limitations in this study, including the relatively small sample size of isolates, the reliance solely on in vitro synergy testing to suggest potential combination therapies, and the absence of experimental validation for the in silico analysis of gene function. Further in-depth studies with a larger pool of isolates are required to widen our knowledge about the newly emerged *A. baumannii* clones in Egypt. Moreover, future in vivo trials are needed to confirm the potential synergistic action of antibiotic combinations with NAC against MDRAB.

## Supplementary Information

Below is the link to the electronic supplementary material.

Supplementary Material 1. Core genome phylogenetic tree of ST85 (GC9) *A. baumannii* clinical isolates. Leaves of the tree are labeled with strain codes. The labels highlighted in yellow indicate the isolates included in this study. *The labels with asterisks represent the strains collected from Alexandria, while the other Egyptian strains were obtained from Cairo. The tree was rooted using ST158 as an outgroup according to Fig. 3. The color strips (from left to right) show the country of isolation, KL, and OCL of the isolates. The distribution of rep types among the strains is displayed where the red circles indicate the presence of the corresponding rep type.

Supplementary Material 2. Core genome phylogenetic tree of ST158 (GC10) *A. baumannii* clinical isolates. Leaves of the tree are labeled with strain codes. The labels highlighted in yellow indicate the isolates included in this study. *The labels with asterisks represent the strains collected from Alexandria, while the other Egyptian strains were obtained from Cairo. The tree was rooted using ST85 as an outgroup according to Figure 3. The color strips (from left to right) show the country of isolation, KL, and OCL of the isolates. The distribution of rep types among the strains is displayed where the red circles indicate the presence of the corresponding rep type.

Supplementary Material 3. Antibiotic resistance genes in the 65 *A. baumannii* strains included in the phylogenetic tree screened using RGI, ABRicate, or BLASTN tools.

## Data Availability

Raw short reads were submitted to the Sequencing Read Archive (SRA) under the BioProject number PRJNA1128891 with BioSample accession numbers (SAMN42110407–SAMN42110419). CP159763, CP160130, and CP160032 are the given NCBI accession numbers for the chromosomes of AB11-AUFP, AB37-AUFP, and AB44-AUFP, respectively. The assembled plasmids from the long-read sequencing data were also deposited in the NCBI database and were given the following accession numbers: CP159764 (pAB11-AUFP1), CP159765 (pAB11-AUFP2), CP159766 (pAB11-AUFP3), CP160131 (pAB37-AUFP1), CP160134 (pAB37-AUFP2), CP160033 (pAB44-AUFP1), and CP160034 (pAB44-AUFP2).

## Abbreviations

MDRAB: Multidrug-resistant *Acinetobacter baumannii*
NAC: *N*-Acetylcysteine
WGS: Whole genome sequencing
MGEs: Mobile genetic elements
CRAB: Carbapenem-resistant *A. baumannii*
AMR: Antimicrobial resistance
THT: Triton Hodge test
MHA: Mueller Hinton agar
BSA: Bovine serum albumin
MLST: Multi-locus sequence typing
QRDRs: Quinolone resistance-determining regions
VFDB: Virulence Factors Database
SRA: Sequencing Read Archive
FICI: Fractional inhibitory concentration index
BAL: Bronchoalveolar lavage
OCL: Lipooligosaccharide outer core loci
ESBL: Extended-Spectrum β-Lactamase
MITEs: Miniature inverted-repeat transposable elements

## Data availability

Most data generated or analysed during this study are included in this published article and its additional files. Any extra demanded details are available from the corresponding author on reasonable request.
