# Supplementary material for: Genomic insights into ST85 and ST158 belonging to recently emerged global clones of multidrug-resistant Acinetobacter baumannii isolates from Egypt: in vitro assessment of repurposed drug–antibiotic combinations
Source: Ann Clin Microbiol Antimicrob. 2025 Nov 15;24:63. doi: 10.1186/s12941-025-00829-0 (PMC12619396; doi:10.1186/s12941-025-00829-0)
Supplement: Supplementary file 1 — Supplementary Material 1. Core genome phylogenetic tree of ST85 (GC9) A. baumannii clinical isolates. Leaves of the tree are labeled with strain codes. The labels highlighted in yellow indicate the isolates included in this study. *The labels with asterisks represent the strains collected from Alexandria, while the other Egyptian strains were obtained from Cairo. The tree was rooted using ST158 as an outgroup according to Fig. 3. The color strips (from left to right) show the country of isolation, KL, and OCL of the isolates. The distribution of rep types among the strains is displayed where the red circles indicate the presence of the corresponding rep type. [file 12941_2025_829_MOESM1_ESM.docx]

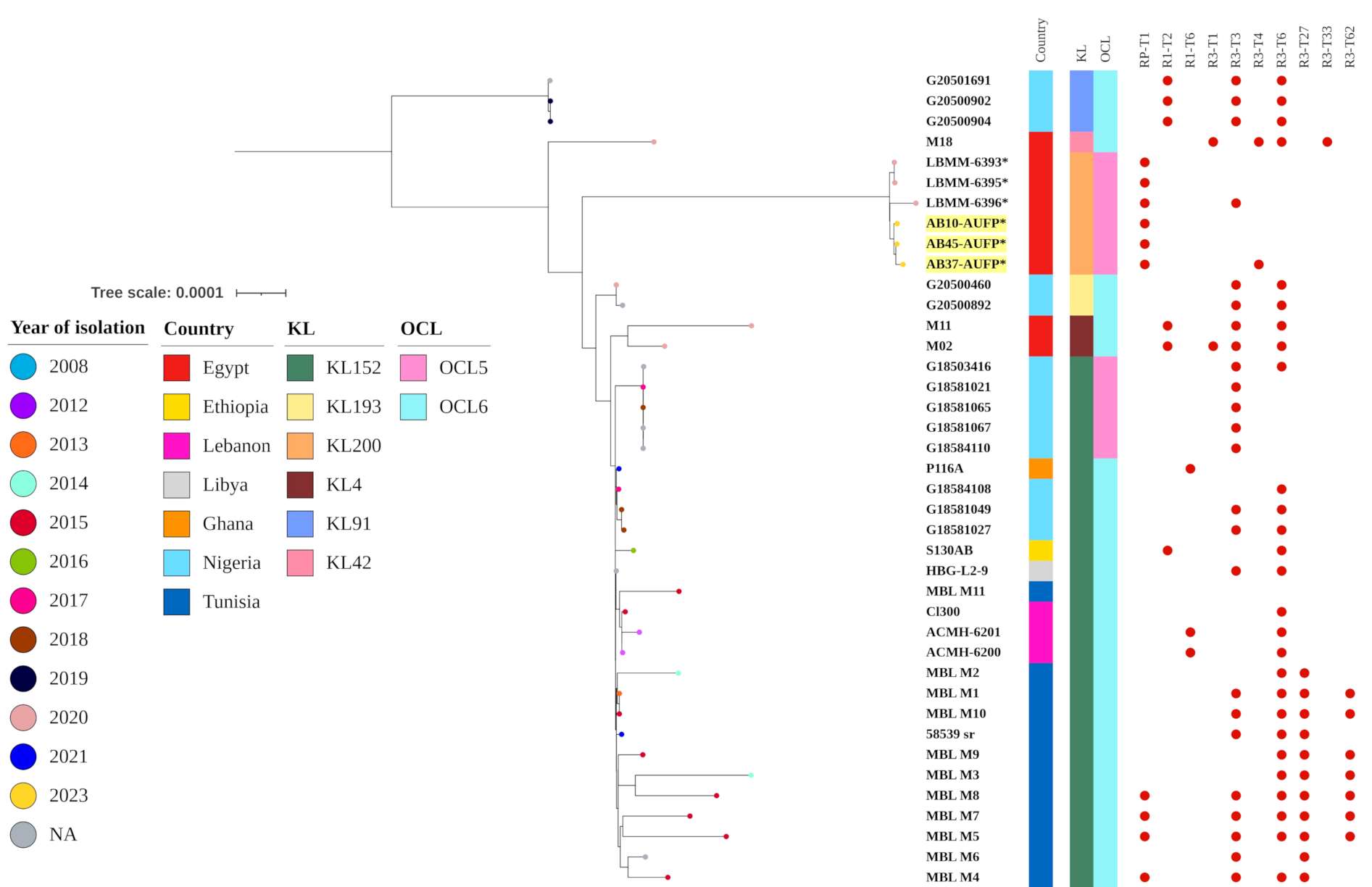


**Additional file 1:** Core genome phylogenetic tree of ST85 (GC9) *A. baumannii* clinical isolates. Leaves of the tree are labeled with strain codes. The labels highlighted in yellow indicate the isolates included in this study. *The labels with asterisks represent the strains collected from Alexandria, while the other Egyptian strains were obtained from Cairo. The tree was rooted using ST158 as an outgroup according to Figure 3. The color strips (from left to right) show the country of isolation, KL, and OCL of the isolates. The distribution of rep types among the strains is displayed where the red circles indicate the presence of the corresponding rep type.
